# Phosphatidic Acid Vesicles Convert the Prion Protein into β-Sheet Fibrils with a Thermostable Structure but Temperature-Sensitive Packing

**DOI:** 10.3390/ijms27198826

**Published:** 2026-10-02

**Authors:** Cyntia Alves, Gabriela Assis-de-Lemos, Michel W. Jaworek, Guilherme A. P. de Oliveira, Roland Winter, Jerson Lima Silva, Tuane C. R. G. Vieira

**Affiliations:** 1 Programa de Biologia Estrutural, Instituto de Bioquímica Médica Leopoldo de Meis, Instituto Nacional de Ciência e Tecnologia de Biologia Estrutural e Bioimagem (INBEB), Universidade Federal do Rio de Janeiro (UFRJ), Rio de Janeiro 21941-599, Brazil; cyntia.alves@bioqmed.ufrj.br (C.A.); gaugusto@bioqmed.ufrj.br (G.A.P.d.O.); jerson@bioqmed.ufrj.br (J.L.S.); 2 Physical Chemistry I—Biophysical Chemistry, Department of Chemistry and Chemical Biology, TU Dortmund University, 44227 Dortmund, Germany; michel.jaworek@tu-dortmund.de (M.W.J.); roland.winter@tu-dortmund.de (R.W.); 3 Instituto D’Or de Pesquisa e Ensino IDOR, Rio de Janeiro 22281-100, Brazil; 4 Faculdade de Medicina e Ciências Biomédicas, Algarve Biomedical Center Research Institute (ABC-Ri), Algarve Biomedical Center (ABC), University of Algarve, 8005-139 Faro, Portugal

**Keywords:** aggregation, fibrilization, prion protein, phosphatidic acid, lipid vesicles

## Abstract

Biomolecular cofactors, including nucleic acids and lipids, promote conversion of the cellular prion protein (PrP^C^) into pathogenic assemblies (prion scrapie, PrP^Sc^), yet the mechanistic role of specific membrane lipids in the earliest stages remains unclear. Previous work showed that phosphatidic acid (PA) vesicles interact in vitro with murine recombinant PrP (recPrP) and trigger PrP aggregation without additional cofactors. Here, we dissect the PA-driven conversion pathway by monitoring PrP assembly and aggregate stability using spectroscopy and by comparing full-length recPrP with an N-terminally truncated construct. PA vesicles induced aggregation in both PrP constructs with similar efficiency, indicating that PA-mediated conversion does not require residues 23–89. Thermal stability analyses revealed a thermally resilient β-structured state accompanied by temperature-sensitive, partially reversible ThT binding, which we interpret as remodelling of higher-order packing. Time-resolved measurements showed that assembly reached a plateau within the measurement time resolution at all temperatures tested, with no resolvable lag phase. At plateau, the extent of ThT-positive assembly was greatest under conditions of higher bilayer order, while light scattering and secondary-structure content were comparable across temperatures. PrP alone showed no detectable temperature-dependent secondary-structure changes between 10 and 40 °C, consistent with membrane organization contributing to assembly architecture. Together, these results establish PA vesicles as a tractable model for interrogating cofactor-driven early prion aggregation and support membrane lipids and their physical state as modulators of PrP conversion into β-structured assemblies, with implications for mechanistic studies and screening strategies targeting lipid–protein interactions.

## 1. Introduction

The conversion of cellular prion protein (PrP^C^) into a disease-associated conformer, called prion scrapie (PrP^Sc^), is associated with prion disease pathogenesis [1,2]. PrP^C^ is a glycosylphosphatidylinositol (GPI)-anchored protein (residues 23–231; ~23 kDa) that localizes to membrane microdomains referred to as lipid rafts [3]. PrP^C^ contains an N-terminal region (residues 23–120) that lacks stable secondary structure and includes the octapeptide repeat segment, which is important to some of the PrP physiological functions, and a C-terminal globular domain (residues 121–231) enriched in α-helix (43%) with low β-sheet content (3%) [4,5]. Disease-associated PrP^Sc^ assemblies show increased β-sheet content and can form amyloid fibrils [6]. Many PrP^Sc^ preparations display partial resistance to proteinase K (PK) digestion and increased stability under denaturing conditions, including elevated temperature and chaotropic agents [7,8,9].

PK resistance varies among prion isolates, and PK-sensitive prion assemblies have been reported in variably protease-sensitive prionopathy (VPSPr) [10,11]. This indicates that PK resistance is not a necessary condition for disease development. Several studies propose that PrP populates intermediate states along the fibrillization pathway [12,13]. Some intermediates have been linked to cytotoxicity or infectivity [14,15]. Strain diversity may be interpreted as conformational diversity of PrP assemblies [12]. Environmental variables can bias which assemblies dominate in vitro [16,17]. Thus, elucidating the mechanisms of structural conversion, the different stages of PrP^Sc^ formation, and the characteristics of the resulting amyloid fibrils are of immense importance for a better understanding of prion diseases.

Biomolecules, including nucleic acids and lipids, may function as cofactors in the conversion of PrP^C^ to PrP^Sc^ by lowering the energetic barrier between the native and pathogenic forms, thereby modulating infectivity and toxicity [18,19,20,21]. Phospholipids with distinct headgroup charges were shown to bind recombinant PrP (recPrP) and promote aggregation (see more in [22]). Wang et al. (2010) showed that POPG (palmitoyl oleoyl-phosphatidylglycerol) combined with RNA promotes the formation of PK-resistant recPrP assemblies that seed endogenous PrP^C^ and cause disease after inoculation in mice [23]. Deleault et al. (2012) reported that synthetic phosphatidylethanolamine (PE) supports recPrP conversion in PMCA in the absence of other added cofactors and that the products are infectious and partially PK-resistant [24,25]. In contrast, Srivastava and Baskakov (2015) did not detect major structural changes in recPrP using biophysical methods upon interaction with PE vesicles [26]. This discrepancy leaves unresolved how specific lipids and the membrane’s physical state shape early conversion steps and which PrP regions are required. Using spectroscopic measurements, we previously showed that phosphatidic acid (PA) vesicles interact with murine recPrP (MuPrP) and induce aggregation in vitro [27].

In the present study, we characterize the PA-driven conversion pathway. First, we test whether the PrP N-terminal region is required for PA-induced aggregation by comparing full-length and N-terminally truncated recPrP. We then test whether the physical state of the PA bilayer modulates PrP assembly by varying temperature as an experimental handle for bilayer order and conformational dynamics. We monitor fibril formation and aggregate stability using spectroscopy and thermal stability measurements. Our results support PA-driven fibrillization that proceeds through β-sheet–rich intermediates with high thermal stability. Fibrillization is favored under conditions consistent with increased bilayer order, supporting a model in which PA membrane organization contributes to conversion efficiency.

## 2. Results

### 2.1. Biophysical Signatures of PrP Assembly in the Presence of Phosphatidic Acid Vesicles

**Figure 1:**
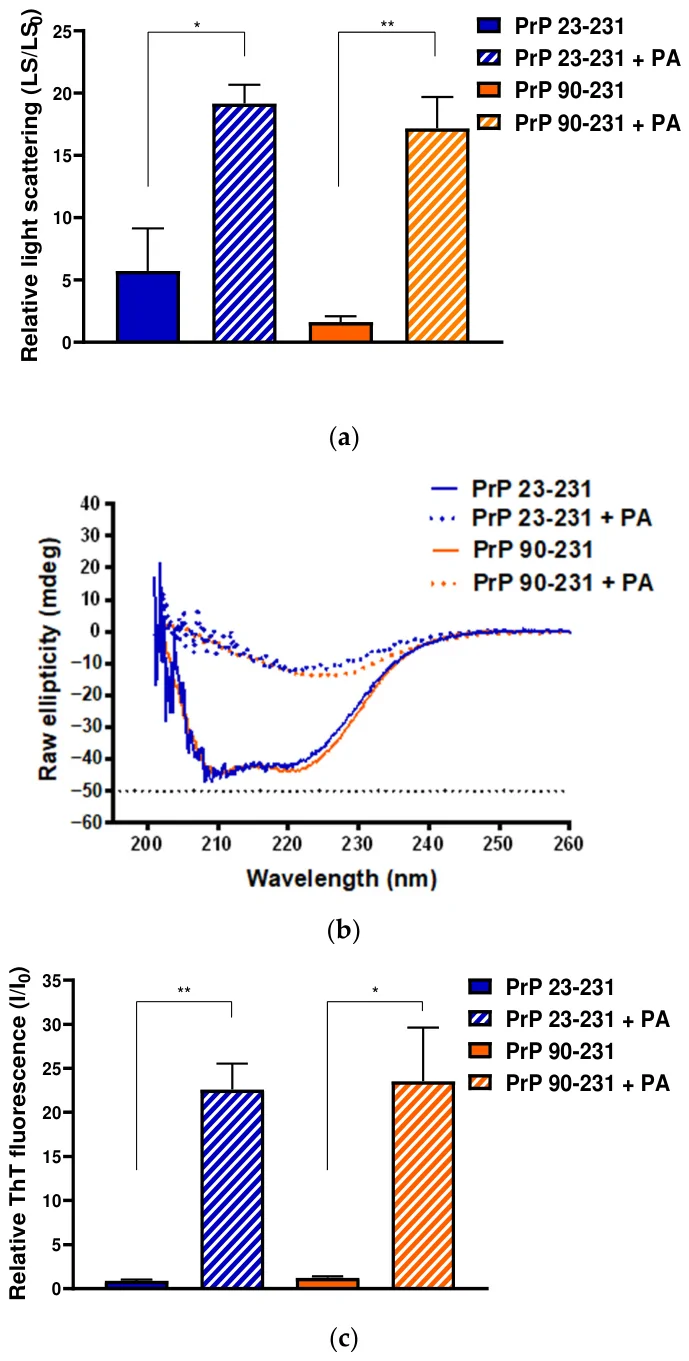
PA promotes PrP aggregation and formation of ThT-positive assemblies. (**a**) Relative light scattering (LS/LS_0_) of recPrP_23–231_ and recPrP_90–231_ in the absence (blue and orange solid bars, respectively) or presence of PA vesicles (blue and orange hatched bars, respectively). (**b**) Representative far-UV CD spectra (raw ellipticity, mdeg) of recPrP_23–231_ and recPrP_90–231_ recorded in the absence (blue and orange continuous lines, respectively) or presence of PA vesicles (blue and orange dotted lines, respectively). (**c**) Relative ThT fluorescence (I/I_0_) of recPrP_23–231_ and PrP_90–231_ in the absence (blue and orange solid bars, respectively) or presence of PA vesicles (blue and orange hatched bars, respectively). Bars show mean ± SD from *n* = 3 independent experiments. Statistics: unpaired *t*-test with Welch’s correction comparing PrP vs PrP+PA within each construct; (* *p* < 0.05, ** *p* < 0.01). Graphs and statistical analysis were generated using GraphPad Prism (version 9.3.0; GraphPad Software, San Diego, CA, USA).

In a previous study, we showed that PA vesicles interact with recPrP and promote aggregation [27]. Here, we characterized PA-induced assemblies formed by full-length PrP_23–231_ and N-terminally truncated PrP_90–231._ Static light scattering (LS) increased upon addition of PA to both PrP_23–231_ and PrP_90–231_ (Figure 1a), consistent with the formation of larger species in solution. No statistically significant differences were observed between the two PrP constructs. To assess the secondary structure of these aggregates, we performed Far-UV CD analysis (Figure 1b). The CD spectral changes in the presence of PA are consistent with a redistribution of secondary structure to a β-sheet-enriched structure when interacting with PA vesicles. We then asked whether these aggregates could interact with ThT, which indicates the formation of amyloid fibrils. As shown in Figure 1c, ThT fluorescence increased strongly for both PrP_23–231_ and PrP_90–231_ in the presence of PA. Together, these data indicate that PA promotes ThT-positive aggregation for both constructs, suggesting that most of the N-terminal region is not required for PA-driven PrP assembly under our experimental conditions. 

### 2.2. Temperature-Dependent Loss of ThT Binding Without Major CD Changes

**Figure 2:**
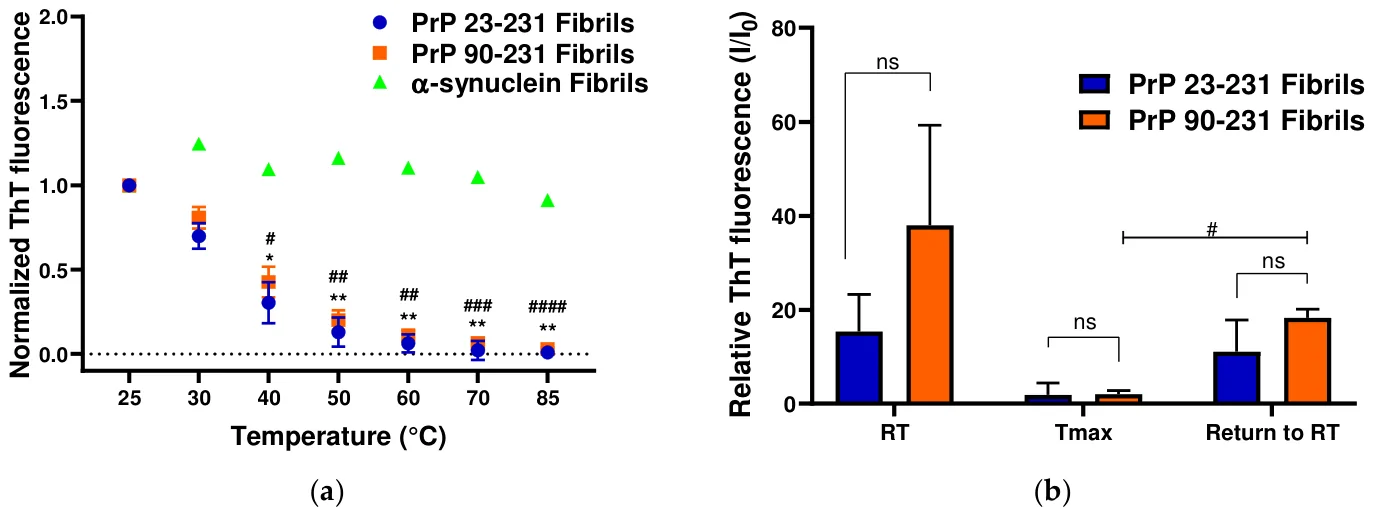

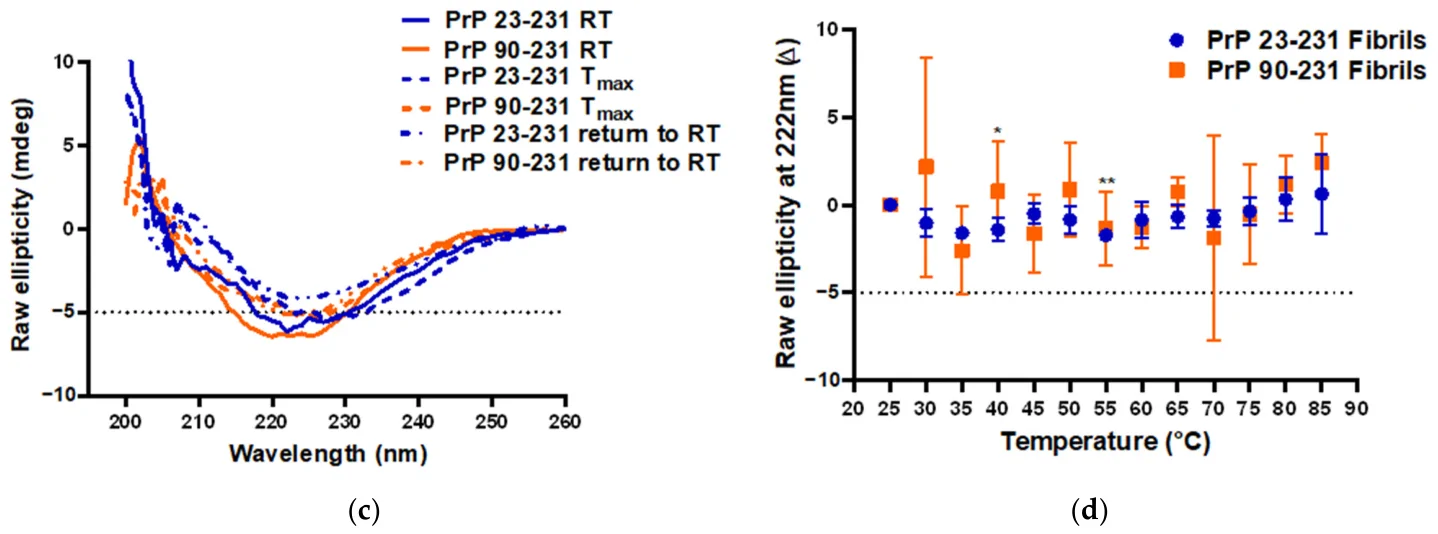
Analysis of PrP:PA fibrils’ structural changes with increasing temperature. (**a**) ThT fluorescence of PrP_23–231_ (blue circles) and PrP_90–231_ (orange squares) fibrils as a function of temperature, normalized to the room temperature signal (25 °C). α-Synuclein fibrils (green triangles) were measured under the same protocol as a control. (**b**) ThT fluorescence of PrP_23–231_ fibrils (blue bars) and PrP_90–231_ fibrils (orange bars) at 25 °C (room temperature, RT), 85 °C (T_max_), and after returning to room temperature (RT), normalized to the room temperature condition. (**c**) Representative far-UV CD spectra of PrP_23–231_ (blue) and PrP_90–231_ (orange) fibrils at RT (continuous lines), T_max_ (dashed lines), and after return to RT (dotted lines). Bars/points represent mean ± SD from *n* = 3 independent experiments. (**d**) Raw ellipticity variation (∆) at 222 nm during temperature increase for PrP_23–231_ (blue circles) and PrP_90–231_ fibrils (orange squares). The secondary structure of both fibrils is temperature-resistant. Statistics—Panels (**a**,**d**): repeated measurements one-way ANOVA followed by Dunnett’s multiple comparisons test, comparing each temperature to the initial point. Panel (**b**): comparisons between constructs were performed using an unpaired *t*-test with Welch’s correction, applying the Holm–Šídák method for multiple comparisons correction. For intra-construct analyses (temperature comparisons), one-way ANOVA followed by Tukey’s multiple comparisons test. Graphs and statistical analyses were generated using GraphPad Prism (version 9.3.0; GraphPad Software, San Diego, CA, USA). For PrP23–231: * *p* < 0.05, ** *p* < 0.01; for PrP90–231: ^#^ *p* < 0.05, ^##^ *p* < 0.01, ^###^ *p* < 0.001, ^####^ *p* < 0.0001, ns = not significant.

To evaluate the thermal response of PrP:PA fibrils, we isolated the fibril-enriched pellet fraction by centrifugation and monitored ThT fluorescence as the temperature increased from 25 °C to 85 °C. ThT signal decreased with temperature for both PrP_23–231_ and PrP_90–231_ fibrils, with no difference between constructs (Figure 2a). To test whether this decrease reflects a general temperature effect on ThT under our measurement conditions, we performed the same experiment with α-synuclein fibrils (Figure 2a). In this control, the ThT signal remained comparatively stable across the temperature range (Figure 2a), indicating that the temperature-dependent loss of ThT signal is specific to the PrP:PA fibril preparations in this assay. We then evaluated whether the loss of the ThT signal coincided with major changes in secondary structure. The ellipticity at 222 nm did not change with temperature for either construct (Figure 2c), and the far-UV CD spectra retained a minimum in the ~220–225 nm region during heating (Figure 2d), showing a temperature-resistant secondary-structure signature consistent with β-sheet enrichment. Finally, after cooling the samples back to 25 °C, the ThT signal partially recovered (Figure 2b), indicating that the ThT-binding arrangement is temperature-sensitive and can be restored at lower temperatures. Together, these observations indicate that the assemblies retain a β-sheet-rich secondary structure while their ThT-binding properties change reversibly with temperature. We interpret this as reversible rearrangement of higher-order packing that modulates dye accessibility or binding, an interpretation supported by the invariance of the 222 nm signal and by the rapid partial recovery of ThT fluorescence upon cooling. This behavior was observed for both PrP_23–231_ and PrP_90–231_, indicating that most of the PrP N-terminal region is not required for this assembly. Given that both PrP constructs fibrillize in the presence of PA and exhibit similar behavior across temperature variations, we focused the subsequent analyses on a single construct: the full-length protein.

### 2.3. PrP:PA Fibrillization Is Temperature Dependent

**Figure 3:**
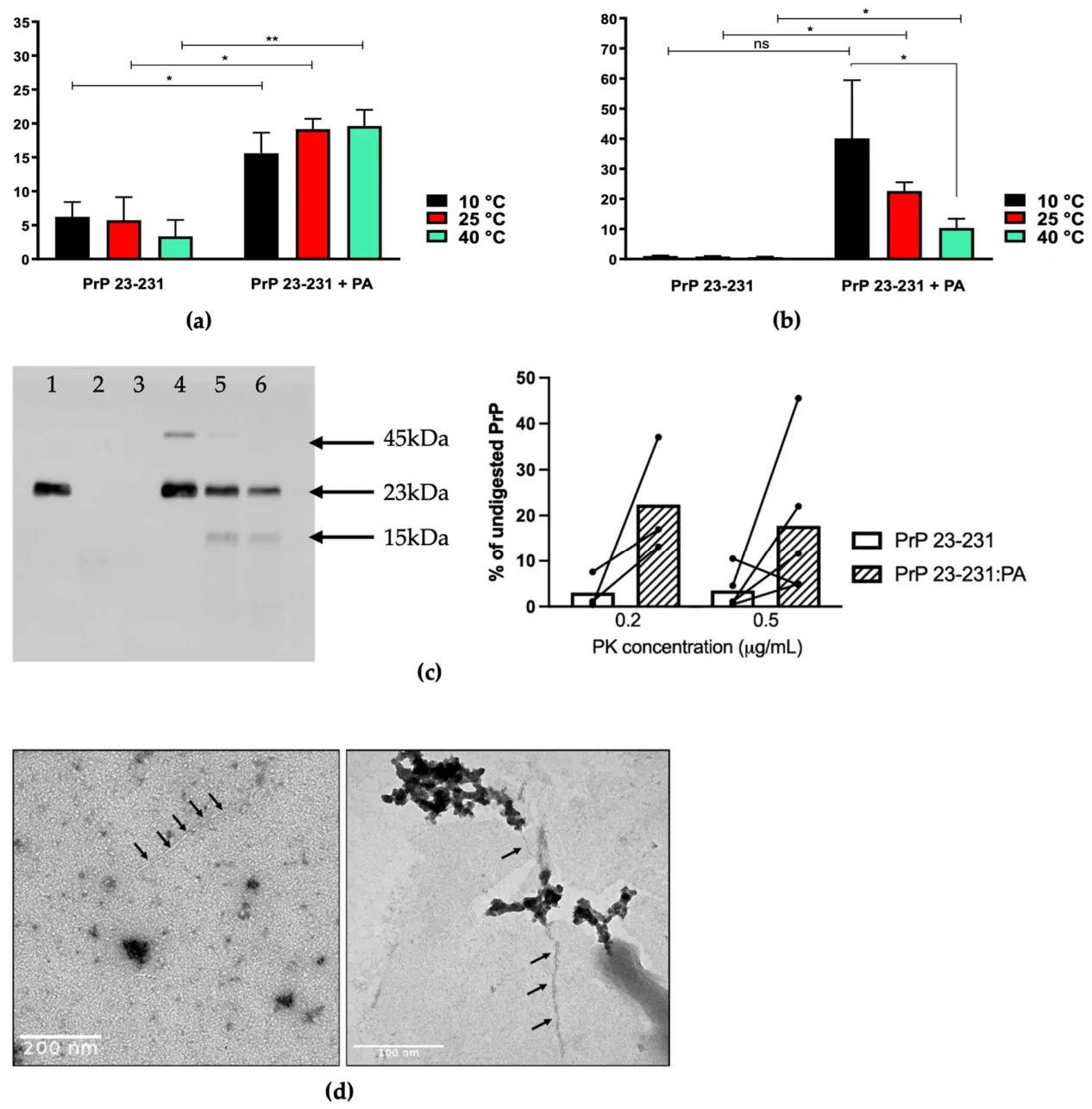
PA-induced formation of ThT-positive assemblies is favored at lower temperatures. (**a**) Relative LS (LS/LS_0_) of PrP_23–231_ in the absence or in the presence of PA, prepared and measured at 10 °C (black), 25 °C (red), or 40 °C (blue). (**b**) Relative ThT fluorescence (I/I_0_) of PrP_23–231_ in the absence or in the presence of PA, at 10 °C (black), 25 °C (red), or 40 °C (blue). For (**a**,**b**), ns = not significant; * *p* < 0.05; ** *p* < 0.01. (**c**) **Left**: Representative immunoblot probed with the anti-PrP antibody 6D11 showing PrP_23–231_ (lanes 1–3) and PrP_23–231_:PA assemblies (lanes 4–6), untreated (lanes 1 and 4) or treated with 0.2 µg/mL (lanes 2 and 5) or 0.5 µg/mL (lanes 3 and 6) proteinase K. Arrows indicate molecular weight markers; note the additional 6D11-reactive band near 45 kDa present only in PA-containing samples, which is greatly reduced by PK treatment. **Right**: Densitometric quantification of band intensities, expressed as the percentage of the corresponding untreated control for each condition. Bars show the mean; symbols show individual independent experiments, with lines connecting paired measurements from the same experiment (*n* = 3 at 0.2 µg/mL, *n* = 5 at 0.5 µg/mL). Assemblies retained, on average, 12.5-fold (0.2 µg/mL) and 6.4-fold (0.5 µg/mL) more signal than the monomeric protein. Ratio-paired *t*-test: *p* = 0.131 at 0.2 µg/mL and *p* = 0.053 at 0.5 µg/mL. (**d**) Representative transmission electron microscopy images (magnification 21,000×) of PrP_23–231_:PA aggregates formed at 10 °C. **Left**: 140 µM PrP with 500 µM PA (1:3.5 molar ratio), used to improve visualization of the fibrillar structures. **Right**: 10 µM PrP with 250 µM PA (1:25 molar ratio), matching the molar ratio used in all spectroscopic experiments. Arrows indicate representative fibrillar structures. Bars show mean ± SD from *n* = 3 independent experiments for panels A and B, and *n* = 3 and *n* = 5 independent experiments for 0.2 and 0.5 µg/mL PK, respectively, in panel C. Statistics—Panels A and B: unpaired *t*-test with Welch’s correction comparing PrP vs PrP+PA within each temperature, with Holm–Šídák multiple-comparison correction method; repeated measurements one-way ANOVA comparing temperatures within each condition, followed by Tukey’s multiple comparisons test. Graphs and statistical analysis were generated using GraphPad Prism (version 9.3.0; GraphPad Software, San Diego, CA, USA).

**Figure 4:**
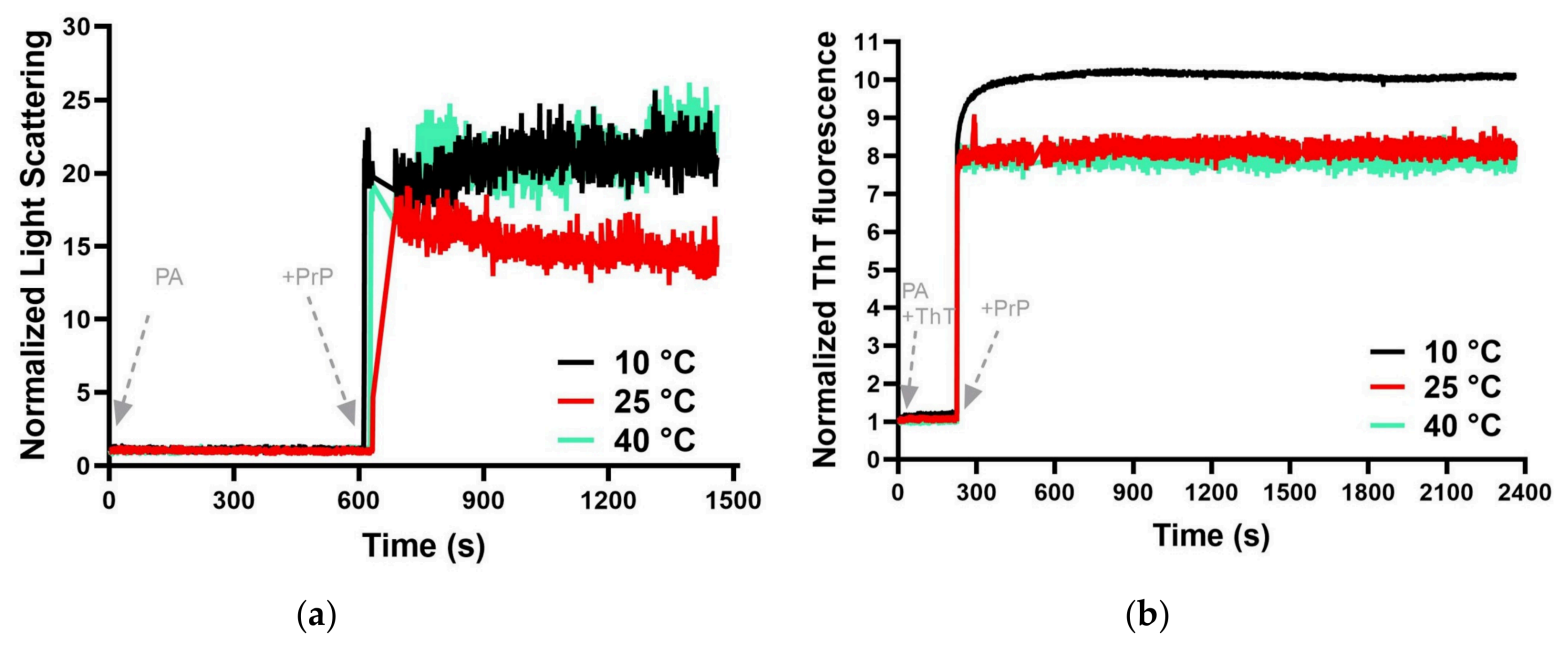
Time-resolved assembly of PrP_23–231_ in the presence of PA vesicles. (**a**) Normalized light scattering and (**b**) normalized ThT fluorescence recorded continuously at 10 °C (black), 25 °C (red) and 40 °C (green). Arrows indicate the addition of PA vesicles (with ThT in (**b**)) and of PrP23–231. Signals are normalized to the baseline recorded in the presence of PA vesicles (and ThT, in (**b**)) prior to the addition of protein. Both signals rise to a plateau within the measurement time resolution at all three temperatures, with no resolvable lag or elongation phase, and remain stable thereafter. The ThT plateau is highest at 10 °C and lowest at 40 °C, whereas light scattering reaches comparable values across the range. Curves are representative of two independent experiments.

To test whether temperature modulates PA-driven assembly, PrP and PA aliquots were equilibrated at 10 °C, 25 °C, or 40 °C, mixed at the target temperature, and measured at the same temperature. In the absence of PA, PrP_23–231_ showed low LS at all temperatures tested (Figure 3a). In contrast, the addition of PA increased LS under all conditions, indicating the formation of larger species in solution. The magnitude of the LS increase was similar across 10–40 °C (Figure 3a). FTIR experiments revealed similar secondary structures of fibrils formed at 10 °C and 40 °C, with increased β-sheet content consistent with amyloid-like PrP secondary structure (Table 1), and no temperature-dependent changes in secondary structure. Consistent with FTIR, Far-UV spectra of PrP23–231 alone or in the presence of PA were similar among all temperatures tested (Figure S1). We then assessed the formation of ThT-positive assemblies. PrP alone showed minimal ThT fluorescence at all temperatures, whereas PrP mixed with PA displayed robust ThT fluorescence (Figure 3b). Notably, the ThT signal tended to be higher at 10 °C and decreased at 25 °C and 40 °C, indicating that lower preparation temperatures may favor the formation of ThT-positive PrP assemblies in the presence of PA (Figure 3b). We note, however, that light scattering was comparable across temperatures (Figure 3a and Figure 4), indicating that similar amounts of large species had formed and that the β-sheet content of the aggregates was equivalent at 10 °C and 40 °C (Table 1). To further characterize these assemblies, Proteinase K (PK) digestion assay was performed. Densitometric quantification showed that assemblies formed in the presence of PA retained a protease-resistant fraction under conditions in which the monomeric protein was largely digested (Figure 3c). At 0.2 µg/mL PK, 22.4 ± 12.8% of the signal remained for PrP:PA assemblies compared with 3.1 ± 3.9% for PrP alone (*n* = 3); at 0.5 µg/mL, the corresponding values were 17.7 ± 17.0% and 3.5 ± 4.2% (*n* = 5). Expressed as the ratio between paired measurements within each experiment, the assemblies retained on average 12.5-fold and 6.4-fold more signal than the monomeric protein at the two PK concentrations. This difference did not reach statistical significance with the present number of replicates (ratio-paired *t*-test, *p* = 0.131 and *p* = 0.053), reflecting substantial variation in the magnitude of the effect between preparations; the direction of the effect was nonetheless consistent, with all three paired comparisons at 0.2 µg/mL and four of five at 0.5 µg/mL favoring the PA-containing samples. We therefore describe these assemblies as showing partial and variable protease resistance rather than the robust resistance characteristic of PrP^Sc^. Immunoblots of the PA-containing samples also consistently showed an additional 6D11-reactive band migrating above the monomeric protein, which was never observed in PrP alone (Figure 3c, lane 4) and was greatly reduced by PK treatment. We refrain from assigning a molecular species to this band: its apparent mass is compatible with a dimer, but SDS-PAGE migration of lipid-containing samples is not reliably related to molecular mass, and we cannot exclude that it corresponds to a monomer whose migration is retarded by residual lipid. This observation establishes that assembly in the presence of PA generates an SDS-stable species that the monomeric protein does not form under the same conditions. In agreement with these findings, transmission electron microscopy (TEM) analysis revealed the presence of higher-order aggregates and filamentous structures (Figure 3d), corroborating the PrP fibrillization observed in the spectroscopic experiments. Interestingly, fibrils formed at 25 °C displayed a morphology similar to those formed at 10 °C (Figure S2). Grids were prepared principally at a PrP:PA ratio of 1:3.5, which improves visualization of the fibrillar structures; a grid prepared at the 1:25 ratio used in the spectroscopic experiments showed comparable filamentous morphology (Figure 3d), indicating that the structures visualized are representative of the assemblies characterized spectroscopically. Together, these results indicate that temperature influences the extent of PrP fibrillization and aggregate stability in the presence of PA, while preserving the overall fibrillar morphology of the assemblies formed under different conditions.

### 2.4. Assembly Plateaus Within the Measurement Time Resolution at All Temperatures

To determine whether the endpoint measurements reported above correspond to a completed reaction and to establish whether the temperature dependence of the ThT signal reflects the rate or the extent of assembly, we monitored light scattering and ThT fluorescence continuously at 10 °C, 25 °C and 40 °C (Figure 4). At all three temperatures, addition of PrP_23–231_ to PA vesicles produced an immediate rise in both signals to a plateau that was reached within the time resolution of the acquisition and remained stable for the duration of the measurement. No lag phase and no resolvable elongation phase were detected, indicating that assembly under these conditions is complete on a timescale shorter than our sampling interval and that the values reported elsewhere in this work correspond to the plateau of the reaction.

The ThT plateau differed between temperatures, being highest at 10 °C and lowest at 40 °C (Figure 4b). Because all three conditions reach plateau on the same timescale, this difference reflects the extent rather than the rate of ThT-positive assembly. Light scattering, by contrast, reached comparable plateau values across the range and did not follow the ordering of the ThT signal (Figure 4a), indicating that similar amounts of large assembled species form at all three temperatures while their dye-binding organisation differs. These observations parallel the endpoint measurements of Figure 3a,b and are consistent with the equivalent β-sheet content determined by FTIR for aggregates formed at 10 °C and 40 °C (Table 1).

### 2.5. Temperature Modulates the Lateral Organization of PA Bilayers

**Figure 5:**
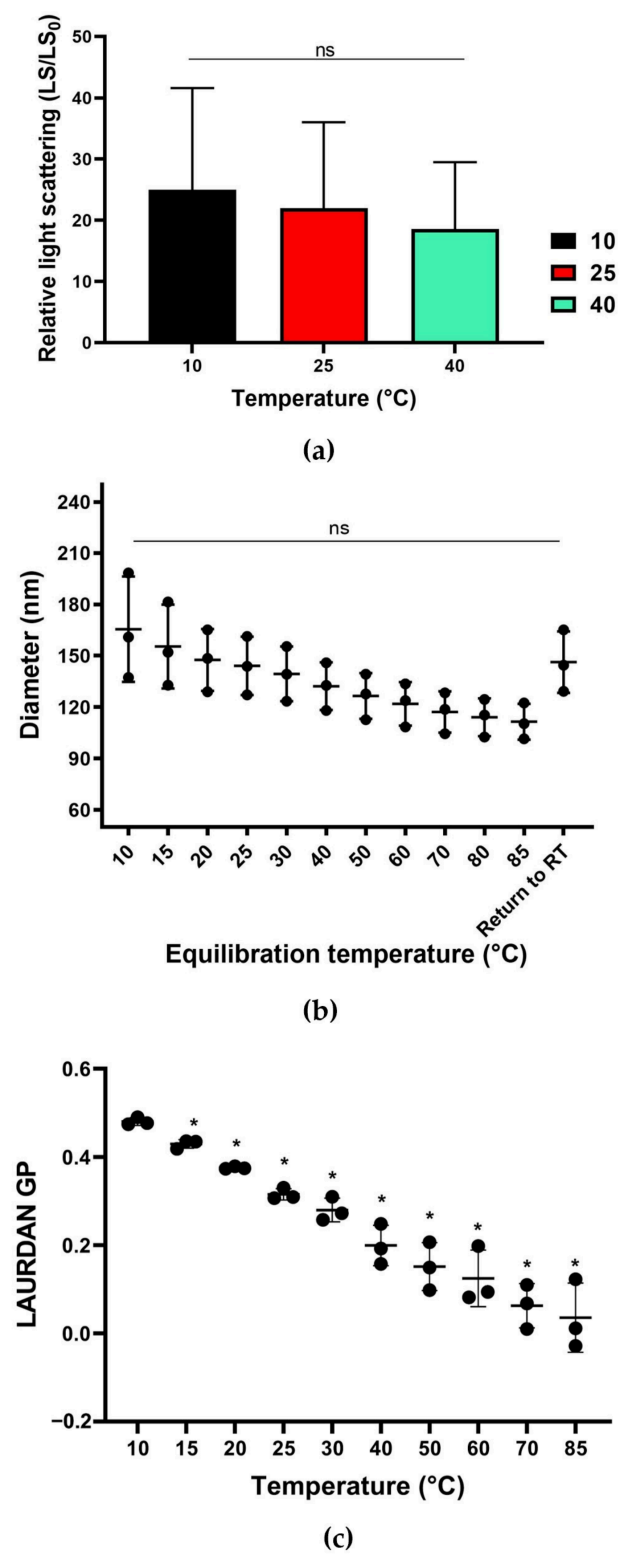
Temperature modulates PA vesicle size and membrane order. (**a**) Relative LS (LS/LS_0_) of PA vesicles prepared at 10 °C (black), 25 °C (red), or 40 °C (green). (**b**) Hydrodynamic diameter (DH) of PA vesicles determined by dynamic light scattering after equilibration at the indicated temperatures. Unlike the light-scattering measurements in (**a**) and the Laurdan measurements in (**c**), which were acquired under active temperature control, DLS acquisition was performed immediately after equilibration without thermal control (see Section 4.2). (**c**) Laurdan generalized polarization (GP) of PA vesicles as a function of temperature. Decreasing GP indicates reduced membrane order under these measurement conditions. Bars/dots show mean ± SD from *n* = 3 independent experiments. Statistics: LS: one-way ANOVA, followed by Tukey’s test for multiple-comparison corrections; DLS/GP: repeated measurements one-way ANOVA followed by Dunnett’s multiple comparison test, comparing each temperature to the initial point; graphs and statistics were generated in GraphPad Prism (ns—not significant, * *p* < 0.05).

Because PrP_23–231_ showed no detectable change in its far-UV CD and FTIR signature between 10 °C and 40 °C (Figure S1 and Table 1), we examined whether temperature alters PA vesicle properties under our experimental conditions. LS measurements of PA vesicles did not show a marked temperature-dependent shift (Figure 5a). Vesicles equilibrated across this range remained within the expected LUV size range, with a modest monotonic trend toward smaller hydrodynamic diameters at higher equilibration temperatures and partial recovery after cooling (Figure 5b and Table S1). Because our DLS instrument lacks active temperature control, we acquired these measurements immediately after equilibration rather than under continuous thermal control and therefore report the direction of the trend rather than absolute dimensions at each temperature (see Methods). Consistent with stable dispersion, the polydispersity index (PDI) remained within the range expected for homogeneous vesicle preparations (Table S1). In contrast, the Laurdan GP value decreased with increasing temperature (Figure 5c), indicating a clear reduction in membrane order (increased bilayer fluidity) in this assay. Therefore, within the tested range, temperature primarily altered the PA bilayer’s lateral organization rather than producing large changes in vesicle size. Notably, reduced membrane order co-occurred with lower PA-dependent ThT signal in the PrP samples (Figure 3b), consistent with the membrane’s physical state of the PA bilayer contributing to the efficiency of forming ThT-positive PrP assemblies.

### 2.6. Cytotoxicity of Fragmented but Not Intact PrP:PA Fibrils

**Figure 6:**
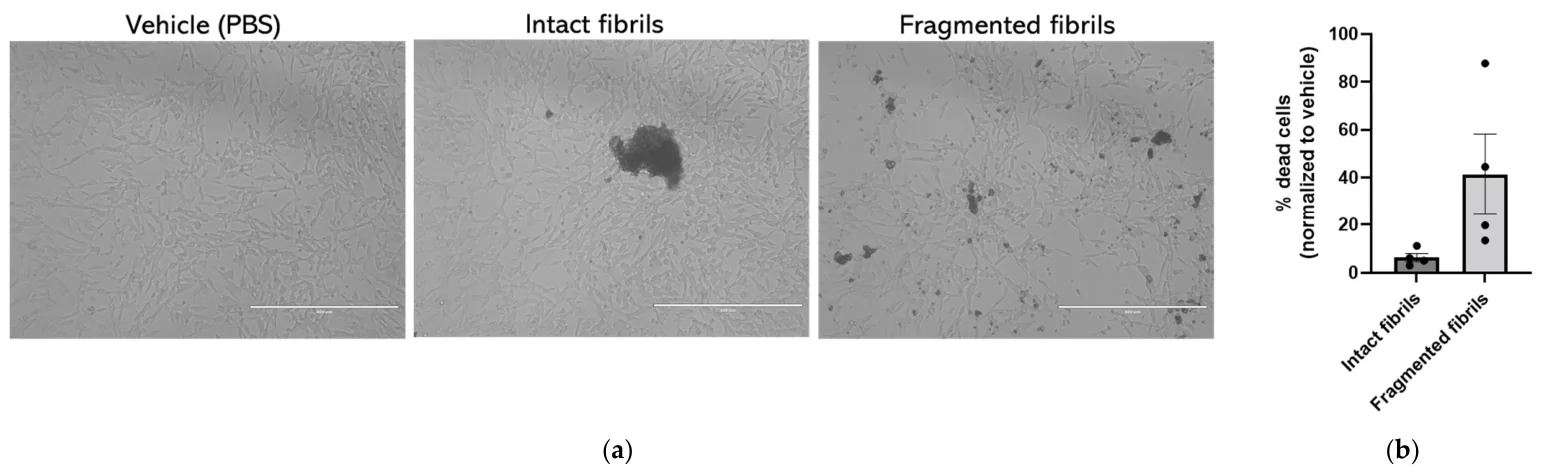
Fragmented, but not intact, PrP:PA fibrils exhibit cytotoxicity toward SH-SY5Y cells. (**a**) Representative bright-field microscopy images of SH-SY5Y cells after 48-h exposure to PBS (vehicle control), intact fibrils, or fragmented fibrils. Both preparations were applied at a final concentration of 3 µM. The dark cluster in the middle panel corresponds to exogenously added PrP:PA fibrillar intact aggregates. Upon sonication, the large aggregates are disrupted into smaller, more diffuse fragments, as shown in the right panel. Scale bar: 400 µm. (**b**) Quantification of the percentage of dead cells after 48-h exposure to intact and fragmented fibrils relative to the vehicle-treated control (set as 0% baseline toxicity). Bars show mean ± SEM from *n* = 4 independent experiments.

After establishing that PA drives PrP assembly into fibrillar aggregates, we next asked whether these fibrils exert cytotoxic effects in cultured cells. Intact and fragmented assemblies were applied to SH-SY5Y cells at a final concentration of 3 µM for 48 h. At this concentration, fragmented assemblies elicited substantial LDH release relative to vehicle-treated controls, whereas intact assemblies did not differ from control (Figure 6). Together, these data show that PrP:PA fibrils are largely inert in their intact state in this assay, whereas fragmentation reveals a cytotoxic potential.

## 3. Discussion

Since PrP was proposed as the agent of transmissible spongiform encephalopathies, many studies have addressed how PrP^C^ converts into disease-associated assemblies. Lipids can modulate recombinant PrP (recPrP) assembly in vitro [22,27], but the specific contribution of defined membrane properties remains incompletely characterized. Here, we investigated PrP assembly in the presence of anionic phosphatidic acid (PA) and evaluated the structural features of the resulting species by spectroscopy. PA vesicles promoted PrP assembly for both the full-length construct (PrP_23–231_) and the N-terminally truncated construct (PrP_90–231_). The similar behavior of these constructs shows that residues 23–89 are not required for PA-driven formation of ThT-positive assemblies in our system. Because PA carries a negatively charged headgroup, electrostatic interactions with positively charged regions of PrP (enriched in lysine and arginine residues) are a plausible contributor to the initial PrP–membrane association [28]. Defining the dominant interaction site(s) and sequence determinants of PA recognition warrants further investigation. This does not exclude contributions from the remaining N-terminal segment present in PrP_90–231_, including positively charged residues within the 90–111 region that have been implicated in conversion in other experimental contexts [29].

Our data provide a picture of PA-driven PrP assembly. PA acts as template and promotes the formation of larger PrP assemblies that exhibit ThT-binding properties and a morphology typically associated with amyloid-like structures. FTIR and CD measurements indicate a secondary-structure profile in the PrP:PA preparations that is consistent with a β-sheet-rich conformation. Together, these results support that PA vesicles constitute a defined, tunable membrane platform that drives PrP assembly into ThT-positive, β-structure-enriched species under controlled conditions.

These findings also motivated specific next steps. The temperature scans highlight a separation between a secondary-structure signature that persists upon heating and a higher-order arrangement that modulates ThT binding and can be partially restored upon cooling. We interpret this behavior as reversible remodeling of packing features that control dye accessibility, without global loss of β-sheet-consistent secondary structure. An alternative interpretation is partial thermal dissociation of the assemblies into smaller oligomeric species with reassociation on cooling. We favor the former because the 222 nm signal remains unchanged throughout the ramp, whereas dissociation would be expected to disrupt the cross-β core, and because the recovery of ThT signal occurs on the timescale of the cooling step, which is short relative to the nucleation-dependent reassembly that dissociation would require. We cannot, however, exclude limited dissociation confined to fibril termini or peripheral chains, which would leave the core intact and would not be resolved by our measurements. This distinction is important because it frames prion conversion as a process in which secondary-structure stabilization and higher-order packing can be uncoupled.

This interpretation requires that the loss of ThT signal not be attributable to the dye itself. ThT is a molecular rotor whose fluorescence quantum yield depends on restriction of torsional motion and is therefore intrinsically sensitive to temperature and to the viscosity of its environment. However, α-synuclein fibrils measured under an identical thermal protocol retained their signal across the same range, indicating that the behavior we observe is a property of the PrP:PA assemblies rather than a general feature of ThT fluorescence at elevated temperature. Whether this is specific to PA-induced assemblies or shared by other in vitro PrP preparations remains unclear, and we are not aware of a systematic comparison across preparations. Relevant evidence indicates, however, that recombinant PrP aggregates differ markedly in the maturity of their higher-order packing: aggregates formed by 2 h of thermal treatment dissociate under pressure with a p½ of 2.8 kbar, whereas those formed over 48 h show no detectable change in their infrared spectra up to 12 kbar, indicating a densely packed structure lacking internal cavities [30]. Because ThT reports on the ordered channels created by higher-order packing rather than on β-sheet content as such, assemblies differing in packing maturity would be expected to differ in the thermal sensitivity of their dye binding. The assemblies characterized here form at a membrane interface and plausibly correspond to a less consolidated packing state.

However, our data do not establish when such a core emerges during assembly. Determining whether it forms before the mature fibril architecture is established will require time-resolved measurements during fibrillization. It also raises an important question: which structural features are most closely linked to biological activity, including cytotoxicity and, in some contexts, propagation?

Fragmentation-dependent bioactivity is frequently reported in amyloid systems, in which smaller oligomeric assemblies or fibril fragments often exert stronger cellular effects than large mature fibrils [31,32]. Consistent with this pattern, fragmented PrP:PA assemblies elicited LDH release at a concentration at which intact assemblies did not, indicating that biological activity is associated with an assembly state generated by fragmentation rather than with fibril presence per se. Because this comparison was made at a single concentration, we cannot determine whether intact assemblies are inactive or merely less potent. Importantly, the PrP:PA assemblies characterized here display β-structure-consistent signatures, yet they show lower PK resistance than typically reported for PrP^Sc^. Several factors distinguish these assemblies from brain-derived PrP^Sc^. The material itself differs: PrP^Sc^ from infected brain is glycosylated, GPI-anchored and propagated through many cycles of templated conversion in vivo, whereas our assemblies form from non-glycosylated recombinant protein at a synthetic membrane interface within 30 min. More specifically, protease resistance depends on the density of higher-order packing that occludes cleavage sites rather than on β-sheet content as such. Our thermal data support the same interpretation: the β-structured core of these assemblies withstands heating to 85 °C, while the ThT-binding organization is temperature-sensitive and partially reversible. An assembly whose β-sheet core has formed while its higher-order packing remains labile is precisely the species expected to show detectable but limited protease resistance, and the limited PK resistance we observe is therefore consistent with, rather than anomalous to, the thermal behavior of these assemblies.

The appearance of an SDS-stable, 6D11-reactive species in PA-containing samples, absent from the monomeric protein, indicates that assembly generates interactions that survive SDS denaturation. Its sensitivity to proteinase K distinguishes it from the SDS-stable species described for PrP^Sc^ and fits the same picture of assemblies in which a β-structured core has formed while higher-order packing remains labile.

Notably, the biological activity of these assemblies does not track their biochemical stability: despite limited protease resistance relative to classical PrP^Sc^ preparations, the fragmented material elicits a cellular response. This positions the PrP:PA system as a controlled in vitro platform to dissect cellular effects associated with PrP assemblies and for examine how stability, fragmentation, and membrane context shape toxic phenotypes.

We emphasise that the biochemical features shared with PrP^Sc^—β-sheet-enriched secondary structure, fibrillar morphology, ThT binding, partial protease resistance and the presence of an SDS-stable species—indicate a PrP^Sc^-like organisation rather than equivalence to PrP^Sc^. The assemblies described here are non-glycosylated, lack a GPI anchor, show only partial and variable protease resistance, and their SDS-stable species is sensitive to proteolysis. We make no claim regarding infectivity, which we have not tested.

Fragmentation is also a central determinant of prion propagation in vivo. In yeast, the Hsp104 disaggregase severs amyloid fibrils to generate the propagons transmitted during cell division, and loss of Hsp104 abolishes [PSI+] inheritance [33,34,35]. Notably, the temperature at which Sup35NM is assembled determines which structural polymorph is formed [36], and it is the frangibility of the resulting aggregate, rather than its rate of growth, that dictates the strength of the prion phenotype: the less stable, low-temperature polymorph divides more frequently and produces a larger number of smaller particles [37]. An inverse relationship between conformational stability and propagation rate has also been described for mammalian prions, where synthetic PrP isolates of lower conformational stability, assessed by guanidine hydrochloride denaturation, showed shorter incubation times [38]. The pattern we observe (assemblies formed at lower temperature showing altered ThT binding and detectable protease resistance, with lower biochemical stability than classical PrPSc preparations, yet acquiring cytotoxicity upon fragmentation) is consistent with this relationship between assembly conditions, structural polymorphism, fragility and biological activity.

The cellular experiments reported here are exploratory. They were performed at a single concentration and with a single readout and therefore establish that fragmentation generates a species capable of compromising membrane integrity without characterizing the concentration dependence of the effect or its mechanism. Notably, these assemblies combine β-sheet-enriched structure with lower biochemical stability than classical PrP^Sc^ preparations, yet their fragmented forms remain biologically active. This dissociation between biochemical stability and cellular activity is the aspect we consider most worth pursuing, and the PrP:PA system provides a tractable setting in which to examine it.

Phospholipids have been implicated as cofactors in the conversion of recPrP in several contexts [22]. POPG combined with RNA has been reported to generate PK-resistant assemblies that propagate and cause disease upon inoculation in mice [39,40]. PE has been reported to support recPrP conversion in PMCA in the absence of other added cofactors [24]. Our PA vesicle system provides an experimentally tractable platform for tuning and measuring membrane properties alongside assembly outcomes. Interestingly, PA is increased in mitochondrial fractions from prion-infected mice [41], suggesting that PA may be a biologically relevant lipid partner that interacts with PrP in vivo and modulates its aggregation behavior.

Our temperature experiments suggest that the membrane’s physical state contributes to PA-driven assembly outcomes. PA vesicles showed temperature-dependent changes in membrane order. These effects occur within the same temperature range where amyloid assembly efficiency changes. This co-variation supports the view that PA bilayer organization and conformational dynamics may bias the formation of amyloid architectures. Time-resolved measurements at 10 °C, 25 °C and 40 °C show that assembly in the presence of PA reaches a plateau within the time resolution of our acquisition at every temperature tested, with no resolvable lag or elongation phase. The values reported here therefore correspond to the plateau of the reaction rather than to an arbitrary point along an ongoing process. Because all three temperatures reach a plateau on the same timescale, the differences we observe between them reflect the extent of ThT-positive assembly rather than the time required to reach it. In cells, PrP^C^ is GPI-anchored and can partition into cholesterol- and sphingolipid-rich membrane microdomains [42]. Prior studies link prion infection to changes in cholesterol homeostasis and membrane properties [43,44,45,46,47]. Other studies suggest that PrP^C^ redistribution to less ordered domains can increase susceptibility to conversion [48]. These observations indicate that membrane context matters across systems.

Importantly, hydrodynamic diameters were determined on samples equilibrated at each temperature and measured without active thermal control, so that partial re-equilibration during acquisition is expected to compress the apparent temperature dependence; these data support the direction of the trend rather than absolute values. Membrane order was assessed independently by Laurdan generalized polarization under direct temperature control. Moreover, we used a natural phosphatidic acid mixture, which reproduces the acyl-chain heterogeneity of biological membranes but does not permit resolving the contribution of individual chain compositions, nor detecting local lipid domains whose properties may differ from the ensemble average reported by Laurdan GP. Our TEM analysis was likewise not designed to compare assembly morphology across lipid-to-protein ratios. Comparing defined synthetic phosphatidic acids that differ in chain length and saturation, together with methods that resolve lateral organization, would allow a more direct attribution of assembly efficiency to specific membrane physical parameters.

Our data define a membrane-parameter axis in which bilayer order influences PrP assembly in a controlled in vitro setting.

## 4. Materials and Methods

### 4.1. Materials

Recombinant Syrian hamster or mouse prion protein constructs spanning residues 23–231 (PrP_23–231_) and 90–231 (PrP_90–231_) were expressed in *Escherichia coli* Rosetta2 (DE3) and purified by immobilized metal affinity chromatography using Ni-NTA Superflow resin (Qiagen GmbH, Hilden, Germany; cat. no. 30450). Protein expression was induced with isopropyl-β-D-thiogalactopyranoside (IPTG; Calbiochem, Merck KGaA, Darmstadt, Germany; cat. no. 420322). Cells were lysed using BugBuster reagent (Novagen, Merck KGaA, Darmstadt, Germany; cat. no. 70584). Phosphatidic acid (PA, L-α-phosphatidic acid, egg yolk, chicken-monosodium salt, Avanti Polar Lipids, Alabaster, AL, USA; cat. no. 840101) was used to prepare vesicles by extrusion. This natural lipid mixture contains high amounts of C16:0 (34,2%), C18:0 (11,5%), C18:1 (31,5%) and C18:2 (18,5%) lipid chains. Because egg-yolk PA is a heterogeneous mixture of molecular species with different fatty-acid compositions, it exhibits a broad gel-to-fluid phase transition rather than a single well-defined transition temperature [49]. Phosphate-buffered saline (PBS; pH 7.4) was prepared using reagents from Sigma-Aldrich (St. Louis, MO, USA). The cytotoxicity of the fibrils was assessed using the LDH Glo-Cytotoxicity Assay from Promega (cat no. J2380). The cell lines present in this study were obtained from Rio de Janeiro Cell Bank (BCRJ; Duque de Caxias, Rio de Janeiro, Brazil, cat. no. 0223).

### 4.2. Methods

#### 4.2.1. Recombinant PrP Purification and Preparation

The recombinant PrP purification was performed as previously described [50]. Briefly, bacteria harboring expression plasmids were grown in LB medium supplemented with 50 μg/mL kanamycin and 34 μg/mL chloramphenicol at 37 °C with shaking. Protein expression was induced with IPTG when the culture reached OD_600_ = 0.8. Cells were lysed using BugBuster reagent, and inclusion bodies were isolated according to the manufacturer’s instructions. The inclusion body pellet was solubilized in 8 M guanidine hydrochloride (GdnHCl) and clarified by centrifugation. The supernatant was purified by immobilized metal affinity chromatography (Ni-NTA) on an ÄKTA system (GE Healthcare Life Sciences, Uppsala, Sweden). Refolding and elution steps followed the referenced protocol framework. Eluted PrP was dialyzed in phosphate buffer. Protein concentration was determined by absorption at 280 nm (extinction coefficients: recPrP_23–231_: 62005 M^−1^cm^−1^ and recPrP_90–231_: 26025 M^−1^cm^−1^ for Syrian hamster; recPrP_23–231_: 63495 M^−1^cm^−1^ for mouse). Proteins were aliquoted and stored at −80 °C. Immediately before each experiment, aliquots were centrifuged using 100 kDa MWCO centrifugal filters (nanosep; Cytiva, Marlborough, MA, USA; cat. no. OD100C35) to remove pre-formed oligomers/aggregates, and protein concentration was re-measured.

α-Synuclein E46K protein fibrils used in ThT analysis were prepared and kindly provided by Mayra de A. Marques, following the protocol described in [51]. Briefly, the monomeric protein purified by size-exclusion chromatography was quantified by absorbance at 280 nm (molar extinction coefficient: 5960 M^−1^cm^−1^). Immediately after purification, the protein was transferred to Eppendorf tubes at a final concentration of 140 μM in Tris–NaCl buffer and incubated at 37 °C under agitation at 600 rpm in a thermomixer for one week. The fibrils formed during this process were stored at room temperature until use.

#### 4.2.2. PA Vesicles Preparation and Analysis

##### Preparation of PA Vesicles

Phosphatidic acid (PA) was dissolved in chloroform to a final concentration of 14.3 mg/mL. An aliquot of this solution was transferred to a glass tube and dried under a stream of nitrogen to form a thin lipid film. The film was stored at −20 °C for at least 24 h before use. For vesicle preparation, the lipid film was hydrated in phosphate buffer (pH 7.4) and incubated in a 70 °C water bath until complete dispersion, ensuring adequate lipid fluidity for efficient vesicle formation. Large unilamellar vesicles (LUVs) were prepared at a final concentration of 2 mM by extrusion in a mini extruder set (cat. no. 61000; Avanti Polar Lipids, Alabaster, AL, USA) and by passing the suspension 20 times through a 100 nm pore-size polycarbonate membrane.

##### Light Scattering Measurements

Static light scattering (SLS or LS) and dynamic light scattering (DLS) were used to assess vesicle size. For SLS, spectra were collected from 300 to 340 nm (excitation at 320 nm) using a 10 mm quartz cuvette in an ISS spectrofluorometer (Champaign, IL, USA) equipped with Peltier temperature control. Samples contained 125 µM PA in phosphate buffer (pH 7.4). Samples were equilibrated to the target temperature, and spectra were acquired at that temperature. For DLS, PA vesicles were prepared at 250 µM in phosphate buffer (pH 7.4) and equilibrated for 10 min at each target temperature in a thermomixer. Samples were transferred immediately to a Particle Size Analyzer (Brookhaven Instruments Corp., Holtsville, NY, USA), with acquisition beginning within 20 s of removal from the bath; this handling interval was kept constant across all conditions. The instrument is not equipped with active temperature control, so these values report the hydrodynamic diameter of vesicles equilibrated at the indicated temperature rather than measurements acquired under continuous thermal control. Partial re-equilibration towards ambient temperature during acquisition is expected to compress the apparent temperature dependence; the data therefore support the direction of the trend rather than absolute dimensions at each temperature. Each measurement comprised five consecutive 1-min runs, from which the mean hydrodynamic diameter was calculated; 3 independent vesicle preparations were analyzed. Variation across consecutive runs within a single measurement did not exceed 30 nm at any temperature and showed no directional trend between 20 °C and 40 °C.

##### Laurdan Generalized Polarization Measurements

Membrane order was assessed using Laurdan (2-dimethylamino-6-lauroyl naphthalene; Molecular Probes, Thermo Fisher Scientific, Eugene, OR, USA). Laurdan was added to the lipid solution at ~0.3 mol% (Laurdan:PA) in chloroform prior to film formation. Laurdan addition and extrusion were performed in the dark. Samples were prepared in PBS (pH 7.4) at 250 µM PA. Laurdan emission spectra were recorded using an ISS spectrofluorometer equipped with a Peltier temperature controller, with excitation at 360 nm and emission monitored at 440 nm and 490 nm. Generalized polarization (GP) was calculated as

$$GP=\frac{I_{440}-I_{490}}{I_{440}+I_{490}}$$

where I_440_ and I_490_ are the emission intensities at 440 nm and 490 nm, respectively. High emission at 440 nm indicates an ordered gel phase, whereas an emission maximum at 490 nm is characteristic for a fluid (liquid-crystalline) lipid phase. Hence, increasing GP-values indicate an increasing lateral order of the lipid acyl chains, while decreasing GP-values indicate increasing disorder and increasing fluidity.

#### 4.2.3. Analysis of PrP Structural Changes upon PA Interaction

To monitor PA-induced aggregation of PrP_23–231_ and PrP_90–231_, we recorded light-scattering (LS) spectra (excitation at 320 nm; emission 300–340 nm). Intrinsic protein fluorescence was measured using excitation at 280 nm and emission collection from 300 to 420 nm. Amyloid fibril formation was monitored by thioflavin T (ThT) fluorescence (excitation at 440 nm; emission 470–520 nm). Measurements were performed in an ISS spectrofluorometer (Champaign, IL, USA) using a 10 mm quartz cuvette. Samples were prepared at a 1:25 PrP:PA ratio in PBS (pH 7.4). Spectra were acquired immediately after brief pipetting homogenization. For the ThT assays, ThT was added to a final concentration of 20 µM.

##### Data Processing of LS and ThT Experiments

For relative light scattering (LS), the area under the curve (AUC) was calculated for each spectrum and expressed as LS/LS_0_. For monomeric PrP, LS corresponds to the AUC of the sample and LS_0_ to the AUC of the buffer under the same conditions. For PrP+PA samples, LS corresponds to the AUC of the mixture and LS_0_ to the AUC of PA alone. For thioflavin T (ThT) fluorescence, spectra acquired in the presence of ThT were corrected by subtraction of spectra obtained in its absence to remove scattering contributions. Relative fluorescence was expressed as I/I_0_, where I represents the corrected AUC of the sample and I_0_ the AUC of ThT alone under matching conditions. For temperature-dependent experiments and bar plots representing room temperature (RT), maximum temperature (85 °C; T_max_), and return to RT, corrected values were normalized to the AUC obtained at 25 °C.

##### Time-Resolved Light Scattering and ThT Fluorescence

Measurements were performed in a Peltier-thermostatted cuvette holder at 10 °C, 25 °C, or 40 °C. PA vesicles (125 µM) were equilibrated at the target temperature in the cuvette, and the baseline was recorded; ThT (20 µM) was included for the fluorescence measurements. PrP23–231 was then added to a final concentration of 5 µM, and the signal was monitored continuously for 40 min. Light scattering was recorded with excitation and emission at 320 nm, and ThT fluorescence with excitation at 440 nm and emission at 485 nm, at an acquisition interval of 1 s. Signals are normalized to the baseline recorded in the presence of PA vesicles and to ThT for the fluorescence measurements, immediately before protein addition. This reference differs from that used for the endpoint measurements, which are expressed relative to ThT alone. Curves are representative of two independent experiments.

#### 4.2.4. Circular Dichroism (CD) Spectroscopy

Far-UV circular dichroism (CD) spectroscopy was used to monitor PrP secondary structure. Measurements were performed on a Chirascan spectropolarimeter (Applied Photophysics, Leatherhead, Surrey, UK), with samples pre-equilibrated at the target temperature prior to acquisition. Temperature-ramping experiments were conducted on a Jasco J-715 spectropolarimeter (Jasco Corporation, Tokyo, Japan). Measurements were carried out using a 0.2 cm pathlength quartz cuvette. CD spectra were recorded from 260 to 190 nm, with a bandwidth of 2 nm. Each spectrum represents the average of three consecutive scans. Samples contained 1:25 PrP:PA ratio in PBS (pH 7.4). Data are presented in millidegrees (mdeg).

CD spectra were baseline-corrected by buffer-only subtraction. For visualization of the curves, all CD values were normalized by subtracting the ellipticity value of the first point measured from all subsequent data points, resulting in curves starting at zero. This transformation only shifted the curves vertically and did not affect their overall shape. Spectra were interpreted only over the wavelength range in which the photomultiplier dynode voltage (HT) remained below 700 V; for PrP:PA samples, this restricted the range to wavelengths above approximately 210 nm. Figure S3 shows HT traces for all samples. We recorded spectra of PA vesicles alone and buffer under identical conditions of lipid concentration, path length, and temperature and show them in the same figure. Temperature-dependent structural changes were monitored by recording ellipticity at 222 nm. For each sample, the ellipticity at each temperature was referenced to the value at room temperature to obtain Δ-values, allowing assessment of relative secondary-structure loss upon heating.

#### 4.2.5. Fourier Transform Infrared (FTIR) Spectroscopy

For the FTIR experiments, 13 identical samples containing 1:25 PrP:PA ratio in PBS (pH 7.4) were prepared and incubated for 30 min at the corresponding preparation temperature, and then centrifuged for 15 min. Supernatants were discarded, and the resulting pellets were pooled and resuspended in a single 1.5 mL tube. The sample was centrifuged again, and the final pellet was lyophilized and used for FTIR analysis.

The lyophilized aggregate pellets were subjected to H/D exchange by resuspension in D_2_O. For this purpose, the PrP variants were previously dialyzed against D_2_O using Amicon Ultra centrifugal filter units (2 mL, 10 kDa molecular weight cutoff) and subsequently lyophilized. Samples were resuspended in 50 mM Tris buffer prepared in D_2_O, and the pD was adjusted to 7.4 (pD = pH + 0.4) by addition of DCl or NaOD. FTIR measurements were performed at 20 °C using a temperature-controlled sample cell with an accuracy of ± 0.2 °C. Secondary structure was assigned from the amide I′ region; the PA ester carbonyl band near 1730 cm^−1^ lies outside this region. Additional details regarding the experimental setup and spectral analysis are described in [52].

#### 4.2.6. Transmission Electron Microscopy (TEM)

Aggregates were formed at 10 °C in PBS buffer (pH 7.4). Grids were prepared principally at 140 µM PrP with 500 µM PA (PrP:PA molar ratio 1:3.5), a condition in which the higher protein content improves visualization of the fibrillar structures by negative staining. To verify that the morphology observed was representative of the assemblies characterized spectroscopically, grids were additionally prepared at 10 µM PrP with 250 µM PA (1:25 molar ratio), the exact condition used in all spectroscopic experiments. Samples were centrifuged (10,000 rpm, 15 min) thirty minutes after preparation, and the pellet was used for grid preparation. Aliquots (4 µL) were applied to copper grids (EMS, Hercules, CA, USA, #CF200-cu), blotted after 1 min, and negatively stained with 2% (*w*/*v*) uranyl acetate for 5 s. Images were acquired on a Philips Tecnai transmission electron microscope operated at 80 kV.

#### 4.2.7. PK Digestion Analysis

PrP_23–231_ samples in the absence or presence of PA (1:25, PrP:PA) were prepared at 10 °C in PBS buffer (pH 7.4). After 30 min of incubation, samples were divided into two equal-volume aliquots and centrifuged (10,000 rpm for 15 min). Pellets were resuspended to the initial volume in each tube, and one aliquot was treated with PK at a final concentration of 1 μg/mL and incubated in a thermomixer at 37 °C for 30 min. The reaction was then stopped by adding the protease inhibitor Pefabloc. Following Pefabloc addition, samples were mixed with SDS sample buffer containing β-mercaptoethanol as a reducing agent and 4 M urea as a denaturing agent. The samples were then heated at 99 °C for 5 min to ensure complete protein denaturation. Equivalent volumes of each sample were loaded onto 18% acrylamide/bis-acrylamide SDS-PAGE gels (1.5 mm thickness) for electrophoresis. Proteins separated by SDS-PAGE were transferred onto nitrocellulose membranes using a Bio-Rad semi-dry blotting system at 25 V and 250 mA for 30 min. Membranes were then blocked with 5% (*w*/*v*) bovine serum albumin (BSA) prepared in TBS-T for 30 min before incubation with the primary antibody. Membranes were incubated overnight at 4 °C with a mouse monoclonal anti-PrP antibody (6D11; BioLegend, San Diego, CA, USA, cat no. 808001) diluted 1:10,000 in blocking buffer, washed six times for 5 min each with TBS-T, followed by incubation with an HRP-conjugated anti-mouse IgG secondary antibody (Cell Signaling Technology, Danvers, MA, USA, cat no. 7076S) diluted 1:5000 for 1 h at room temperature. Immunoreactive bands were visualized by enhanced chemiluminescence (ECL) using a ChemiDoc Imaging System (Bio-Rad, Hercules, CA, USA). Band intensities were quantified using ImageJ.JS (version 0.6.0) and expressed as a percentage of the corresponding untreated control (0 µg/mL PK) for each condition. Three independent experiments were performed at 0.2 µg/mL PK and five at 0.5 µg/mL. Because PrP and PrP:PA samples were processed and resolved on the same gel within each experiment, measurements were treated as paired and analysed with a ratio-paired *t*-test (GraphPad Prism), equivalent to a paired *t*-test; this analysis is appropriate for ratio data and removes the contribution of between-gel variation in loading. Individual experiments are shown in Figure 3c.

#### 4.2.8. Temperature Measurements

Temperature was controlled using Peltier units coupled to both the spectrofluorometer and the CD spectrometer. To evaluate the thermal stability of PrP:PA fibrils, samples were prepared at 25 °C. After 30 min, samples were centrifuged (10,000 rpm/15 min) to separate smaller aggregates from the pellet fraction. The pellet was resuspended in PBS and used for temperature-scan measurements in both the CD and the spectrofluorometer. Spectra were acquired from 25 °C to 85 °C in 5 °C stepwise increments. After acquisition at 85 °C, samples were cooled back to 25 °C, and an additional spectrum was recorded. To test the temperature dependence of PrP:PA aggregation, samples were prepared at 10 °C, 25 °C, or 40 °C. For each condition, aliquots were equilibrated for 3 min in a thermomixer (no mixing) prior to sample preparation. Samples were then transferred to the cuvette and measured immediately at the same temperature used for preparation.

#### 4.2.9. Effect of PrP:PA Fibrils on Cellular Viability

To investigate the cytotoxic potential of PrP:PA fibrils, SH-SY5Y human neuroblastoma cells were used. Recombinant mouse PrP_23–231_ was aggregated in a 1:25 PrP:PA ratio in PBS (pH 7.4). To compare the cytotoxic potential of intact and fragmented fibrils, a portion of the aggregates was sonicated in a water bath (model Q336D, Quimis, Diadema, SP, Brazil) for 6 h to fragment the fibrils. These samples were then added to the culture medium at a final concentration of 3 µM. Cell viability was assessed after 48 h of exposure using the LDH release assay.

##### LDH Release Assay

Cell death was quantified by measuring lactate dehydrogenase (LDH) release into the culture supernatant using a commercial kit (Promega Corporation, Madison, WI, USA; cat. no. J2380) according to the manufacturer’s instructions. Briefly, after the treatment period, the supernatant was collected and incubated with the reaction mixture for approximately 30 min. The reaction was stopped after the adjusted time, and absorbance was measured at 490 nm using a microplate reader. Cytotoxicity was calculated as a percentage of the LDH release from cells exposed to vehicle (PBS 1×).

#### 4.2.10. Statistical Analysis

All statistical analyses were performed in GraphPad Prism (version 9.3.0; GraphPad Software, San Diego, CA, USA). Data are reported as mean ± SD, and the number of independent experiments (*n*) is indicated in the corresponding figure legends. All tests were two-tailed, and statistical significance was set at α = 0.05.

For comparisons between two groups (e.g., PrP vs. PrP+PA within a given construct), unpaired *t*-tests with Welch’s correction were used. When multiple pairwise tests were performed within the same figure panel, *p*-values were adjusted for multiple comparisons using the Holm–Šídák method.

For analysis involving three or more independent groups (e.g., comparisons among preparation temperatures at 10 °C, 25 °C, and 40 °C within the same condition), one-way ANOVA followed by Tukey’s multiple comparisons test was applied.

For temperature-ramp experiments in which the same sample was measured across multiple temperatures (e.g., 25 °C to 85 °C, and return to 25 °C), repeated-measures one-way ANOVA was used, followed by Dunnett’s multiple comparisons test comparing each temperature point to the initial 25 °C condition. For LDH cytotoxicity assays comparing two conditions (intact fibrils versus fragmented fibrils), unpaired *t*-test was used.

## 5. Conclusions

This study establishes phosphatidic acid (PA) vesicles as a defined membrane platform that promotes PrP assembly into amyloid, β-structured species for both full-length PrP_23–231_ and N-terminally truncated PrP_90–231_, indicating that residues 23–89 are dispensable for this outcome in our system. Temperature scans revealed a separation between secondary-structure signatures that remain broadly stable up to 85 °C and a ThT-binding organization that is temperature-sensitive and partially reversible, consistent with remodeling of higher-order packing without a major loss of β-structure. Our measurements do not, however, separate this from limited structural rearrangement or partial dissociation of the assemblies, and this distinction remains open. PA vesicles themselves exhibited temperature-dependent changes in membrane order over the same range, linking the membrane’s physical state to the architecture of the resulting assemblies rather than to the amount of material formed. Importantly, PrP:PA fibrils displayed lower biochemical stability than classical PrP^Sc^ benchmarks in our assays, including reduced PK resistance, yet their fragmented forms elicited measurable biological effects. Together, these findings support the view that membrane context and post-assembly fragmentation can generate PrP species with β-structured features and measurable cytotoxicity, positioning the PrP:PA system as a tractable in vitro platform to investigate PrP assembly states and cellular responses relevant to prion biology.

## Figures and Tables

**Table 1:** Percentage distribution of secondary structures in PrP_23–231_:PA aggregates formed at 10 °C and 40 °C, determined by FTIR. The structural composition was similar across the aggregates, regardless of preparation temperature. The increased β-sheet content is comparable to values reported for PrP^Sc^ (6), although this similarity in secondary-structure composition does not imply equivalence to PrP^Sc^.

Fibrils	Loops (%)	α-Helix (%)	Random Coils (%)	β-Sheets (%)
**PrP 23–231_10 °C**	23.3	20.3	23.6	32.8
**PrP 23–231_40 °C**	23.7	20.9	24.0	31.5

## Data Availability

The original contributions presented in this study are included in the article/Supplementary Materials. Further inquiries can be directed to the corresponding author.

## Supplementary Materials

The following supporting information can be downloaded at: https://www.mdpi.com/article/10.3390/ijms27198826/s1.

## Abbreviations

The following abbreviations are used in this manuscript:

AUC	Area under curve
BCRJ	Rio de Janeiro Cell Bank
BSA	Bovine sérum albumin
CD	Circular Dichroism
D_2_O	Deuterium oxide
DLS	Dynamic Light Scattering
ECL	Enhanced chemiluminescence
FTIR	Fourier-transform infrared spectroscopy
GdnHCl	Guanidine hydrochloride
GP	Generalized polarization
GPI	Glycosylphosphatidylinositol
H/D	Hydrogen/deuterium
HRP	Horseradish peroxidase
IMAC	Immobilized metal affinity chromatography
IPTG	Isopropyl-β-thiogalactopyranoside
LB	Lysogeny broth
LDH	Lactate Dehydrogenase
LS	Light Scattering
LUVs	Large Unilamellar Vesicles
MWCO	Molecular-weight cutoff
PA	Phosphatidic acid
PBS	Phosphate-buffered saline
PDI	Polydispersity index
PE	Phosphatidylethanolamine
PK	Proteinase K
PMCA	Protein misfolding cyclic amplification
POPG	Palmitoyl-oleoyl-phosphatidylglycerol
PrP	Prion protein
PrP^C^	Cellular prion protein
PrP^Sc^	Scrapie prion protein/disease-associated prion protein
recPrP	Recombinant prion protein
RT	Room temperature
SD	Standard deviation
SEM	Standard error of the mean
SH-SY5Y	Human neuroblastoma cell line
SLS	Static light scattering
TEM	Transmission Electron Microscopy
ThT	Thioflavin T
T_max_	Maximum temperature
VPSPr	Variably protease-sensitive prionopathy
