# Supplementary material for: Phosphatidic Acid Vesicles Convert the Prion Protein into β-Sheet Fibrils with a Thermostable Structure but Temperature-Sensitive Packing
Source: Int J Mol Sci. 2026 Oct 2;27(19):8826. doi: 10.3390/ijms27198826 (PMC13665344; doi:10.3390/ijms27198826)
Supplement: Supplementary file 1 [file ijms-27-08826-s001.zip › ijms-4506037-supplementary.pdf]

Table S1. Hydrodynamic diameter (DH) and polydispersity index (PDI) of PA vesicles after equilibration at different temperatures, determined by dynamic light scattering (DLS). DH decreased progressively with increasing temperature. PDI values remained within the expected range for homogeneous vesicle preparations under all conditions tested. Data are mean ± standard deviation of 3 independent preparations.

Equilibration temperature (°C)	Hydrodynamic diameter (nm)	Polydispersity Index (PDI)
**10**	165 ± 30.7	0.122 ± 0.085
**15**	155.4 ± 24.4	0.141 ± 0.071
**20**	147.5 ± 18.0	0.106 ± 0.072
**25**	144.1 ± 17.0	0.091 ± 0.085
**30**	139.9 ± 15.9	0.106 ± 0.052
**40**	132.2 ± 13.9	0.127 ± 0.044
**50**	126.5 ± 13.2	0.120 ± 0.045
**60**	121.9 ± 12.6	0.105 ± 0.050
**70**	117.2 ± 12.0	0.113 ± 0.041
**80**	114.1 ± 11.0	0.113 ± 0.046
**85**	111.5 ± 10.4	0.113 ± 0.038
**Return to RT**	146.3 ± 17.9	0.145 ± 0.079

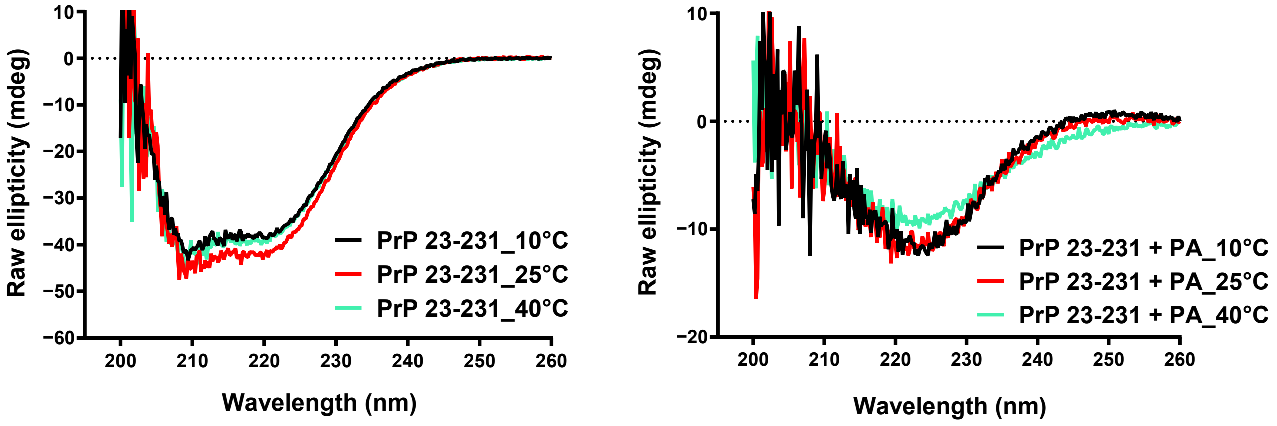

Figure S1. Representative far-UV CD spectra of PrP23-231 in the absence (left) and presence (right) of PA vesicles, acquired at 10 °C (black), 25 °C (red), and 40 °C (green). Samples contained 10 μM PrP and 250 μM PA in PBS (pH 7.4). Curves are representative of 3 independent experiments.

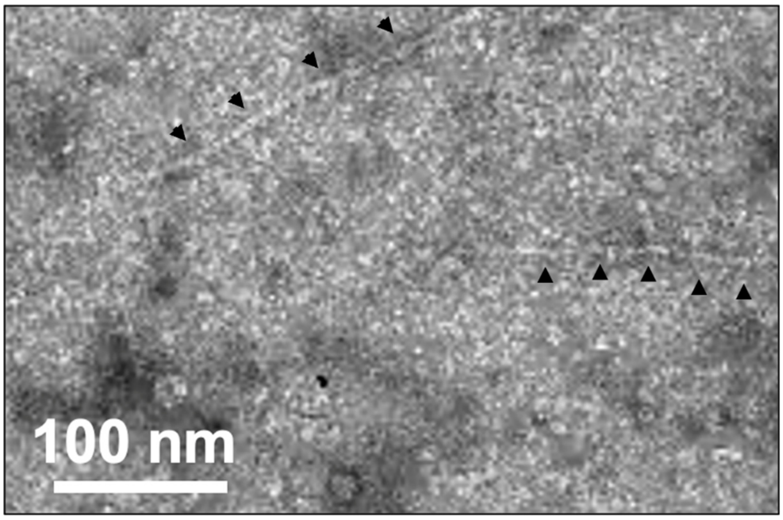

Figure S2. Representative transmission electron microscopy (TEM) image (magnification of 21,000X) of PrP_{23-231}:PA aggregates formed at 25 °C, prepared at 140 µM PrP with 500 µM PA (1:3.5 molar ratio). Scale bar: 100 nm. Image representative of 3 independent preparations.

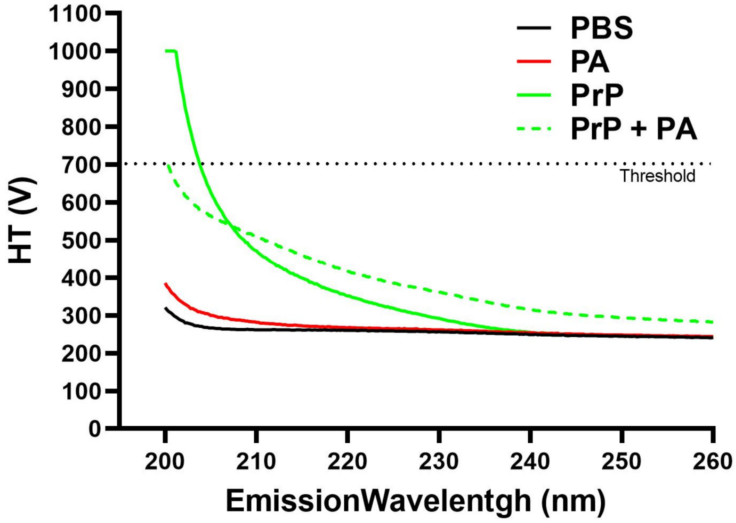

Figure S3. Far-UV CD photomultiplier dynode voltage (HT) for buffer (black), PA vesicles alone (red), PrP23-231 alone (green, solid) and PrP23-231 in the presence of PA (green, dashed), recorded under identical conditions of concentration, path length and temperature. PA vesicles alone produce no measurable CD signal above baseline and show an HT trace indistinguishable from buffer, indicating that the spectral changes reported in Figure 1b arise from the protein component. In PrP:PA samples the HT exceeds 700 V below approximately 210 nm; data below this wavelength were not interpreted. Curves are representative of 3 independent experiments.
